# Supplementary material for: Comparative analysis of gene regulation in single cells using Compass
Source: Cell Rep Methods. 2025 May 8;5(5):101035. doi: 10.1016/j.crmeth.2025.101035 (PMC12146641; doi:10.1016/j.crmeth.2025.101035)
Supplement: Document S1. Figures S1–S3 [file mmc1.pdf]

Cell Reports Methods, Volume *5*

Supplemental information

Comparative analysis of gene regulation

in single cells using Compass

Changxin Wan, Yilong Qu, Zhiyou Ye, Tianbei Zhang, Huifang Ma, Ming Chen, Wenpin Hou, and Zhicheng Ji

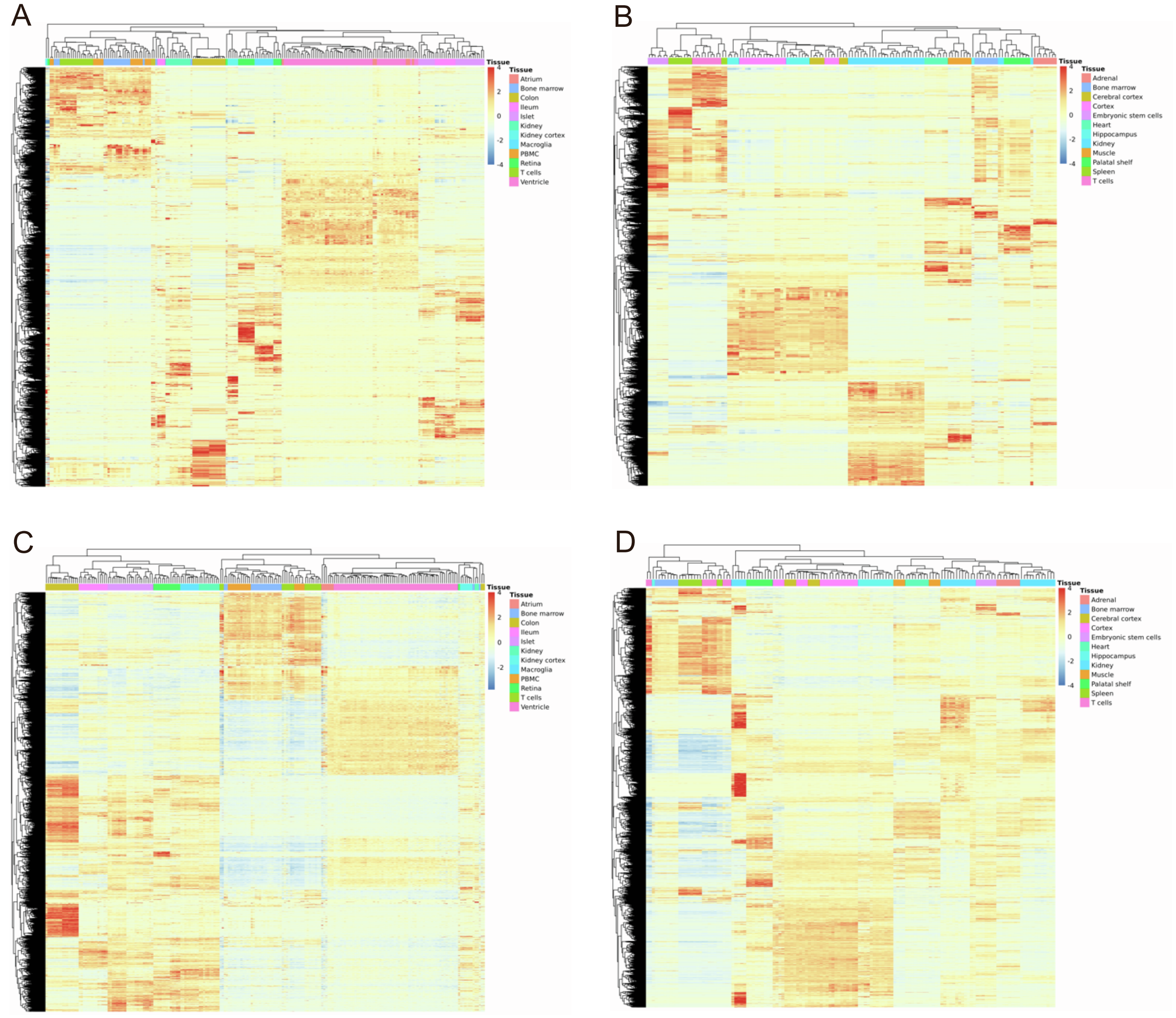

Figure S1. Pseudobulk analysis in CompassDB, related to Figure 2.

(A - B) Gene expression levels of RNA pseudobulks in human (A) and mouse (B) samples. Each row represents a gene and each column represents a sample. Gene expression values are scaled to have a mean of 0 and a standard deviation of 1 for each gene across samples. Samples from different tissues are marked in different colors.

(C - D) Chromatin accessibility levels of ATAC pseudobulks in human (C) and mouse (D) samples. Each row represents a genomic bin and each column represents a sample. Chromatin accessibility values are scaled to have a mean of 0 and a standard deviation of 1 for each genomic bin across samples. Samples from different tissues are marked in different colors.

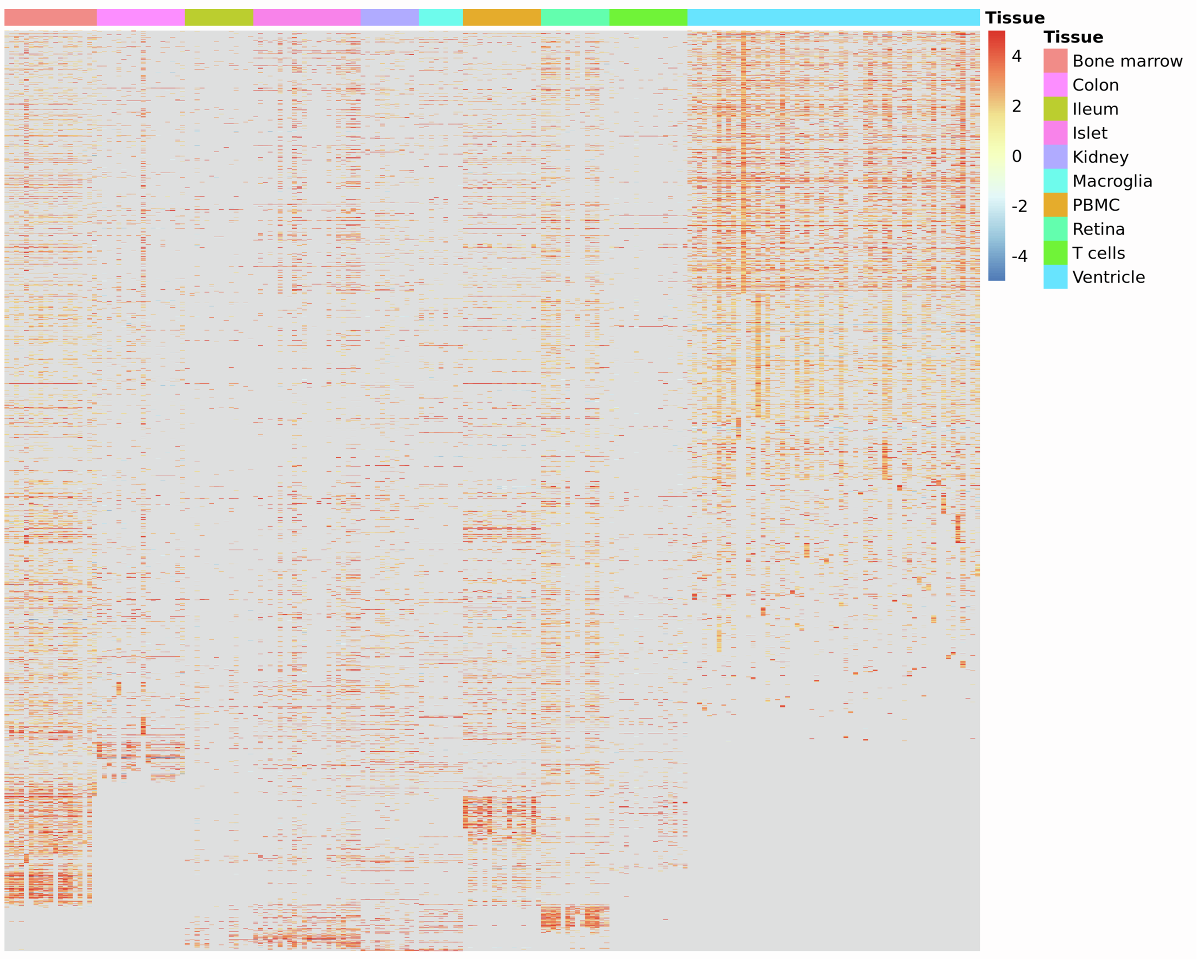

Figure S2. CRE-Gene linkage signal in human samples, related to Figure 2. Each row represents a CRE-gene linkage, and each column represents a sample. If a linkage is present in a tissue, the color indicates the linkage strength values from Signac. Linkages not present in a tissue are shown in gray.

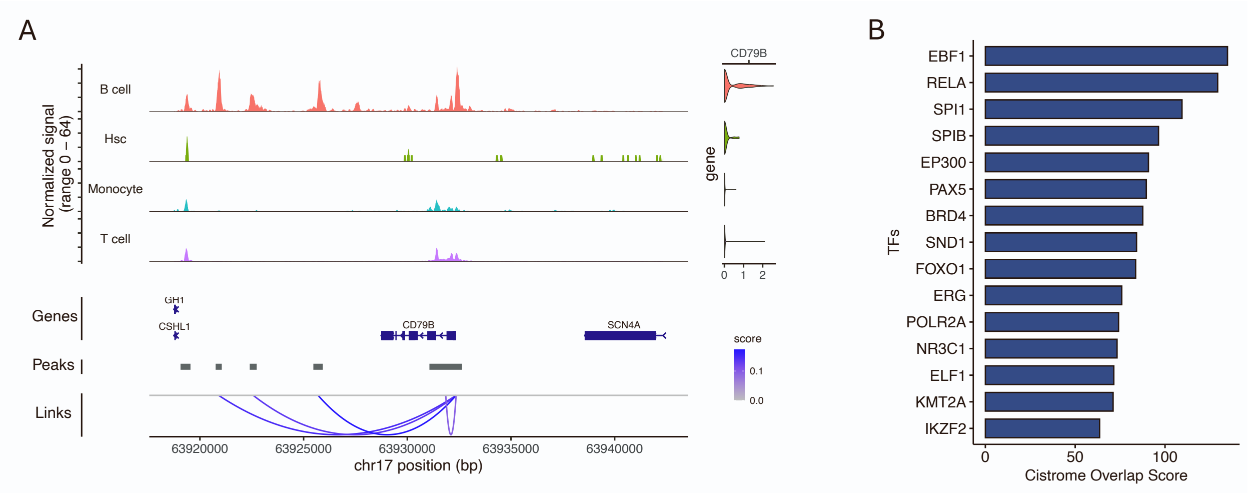

Figure S3. CompassR analysis of the CD79B gene in PBMCs, related to Figure 3. The genome tracks show the normalized ATAC signals in each cell type. Violin plots show expression of CD79B gene in each cell type. Arc plots show CD79B-linked CREs and the color indicates their linkage scores.

(A) CompassR analysis of CD79B gene in a PBMC sample.

(B) TFs enriched in CD79B-linked CREs identified in (A).
