# Supplementary material for: Comparative analysis of gene regulation in single cells using Compass
Source: Cell Rep Methods. 2025 May 8;5(5):101035. doi: 10.1016/j.crmeth.2025.101035 (PMC12146641; doi:10.1016/j.crmeth.2025.101035)
Supplement: Document S2. Article plus supplemental information [file mmc2.pdf]

Cell Reports
Methods

Article

Comparative analysis of gene regulation in single cells using Compass

Graphical abstract

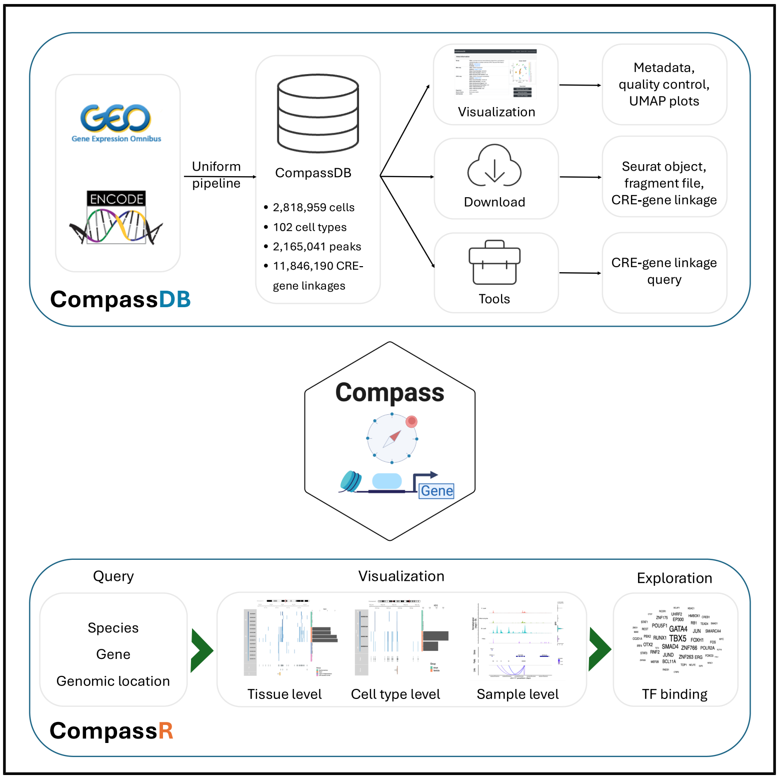

Highlights

- We present Compass, a framework for analyzing gene regulation in single cells
- Compass provides a resource of high-quality, uniformly processed single-cell data
- Compass has robust functions for analyzing CRE-gene linkages
- Advanced visualization capabilities enable comparative analyses of gene regulation

Authors

Changxin Wan, Yilong Qu, Zhiyou Ye, ..., Ming Chen, Wenpin Hou, Zhicheng Ji

Correspondence

zhicheng.ji@duke.edu

In brief

Wan et al. present Compass, a framework for comparative analysis of gene regulation in single cells, which consists of CompassDB and CompassR. CompassDB provides high-quality, publicly available single-cell multi-omics data. CompassR enables the exploration of gene regulation across tissues, cell types, and individual samples.

Wan et al., 2025, Cell Reports Methods *5*, 101035
May 19, 2025 © 2025 The Author(s). Published by Elsevier Inc.
https://doi.org/10.1016/j.crmeth.2025.101035

CellPress

Cell Reports Methods

CellPress
OPEN ACCESS

Article

Comparative analysis of gene regulation in single cells using Compass

Changxin Wan,[1,2] Yilong Qu,[1] Zhiyou Ye,[3] Tianbei Zhang,[1] Huifang Ma,[1] Ming Chen,[4,5] Wenpin Hou,[6] and Zhicheng Ji[1,2,7,*]
[1]Department of Biostatistics and Bioinformatics, Duke University School of Medicine, Durham, NC, USA
[2]Program of Computational Biology and Bioinformatics, Duke University School of Medicine, Durham, NC, USA
[3]Department of Biomedical Engineering, Pratt School of Engineering, Duke University, Durham, NC, USA
[4]Department of Pathology, Duke University School of Medicine, Durham, NC, USA
[5]Duke Cancer Institute, Duke University, Durham, NC, USA
[6]Department of Biostatistics, Columbia University Mailman School of Public Health, New York City, NY, USA
[7]Lead contact
*Correspondence: zhicheng.ji@duke.edu
https://doi.org/10.1016/j.crmeth.2025.101035

MOTIVATION Single-cell multi-omics sequencing, which simultaneously measures chromatin accessibility and gene expression within the same cell, has been widely applied to link *cis*-regulatory elements (CREs) to their target genes. However, most studies focus on a limited number of tissue types, restricting comparative analysis of gene regulation across different tissues. To address this limitation, here we present Compass, a framework that enables the comparison of gene regulation across diverse tissues and cell types by collecting high-quality, uniformly processed, publicly available single-cell multi-omics data.

SUMMARY

Single-cell multi-omics is a transformative technology that measures both gene expression and chromatin accessibility in individual cells. However, most studies concentrate on a single tissue and are unable to determine whether a gene is regulated by a *cis*-regulatory element (CRE) in just one tissue or across multiple tissues. We developed Compass for comparative analysis of gene regulation across a large number of human and mouse tissues. Compass consists of a database, CompassDB, and an open-source R software package, CompassR. CompassDB contains processed single-cell multi-omics data of more than 2.8 million cells from hundreds of cell types. Building upon CompassDB, CompassR enables visualization and comparison of gene regulation across multiple tissues. We demonstrated that CompassR can identify CRE-gene linkages specific to a tissue type and their associated transcription factors in real examples.

INTRODUCTION

Single-cell multi-omics sequencing,[1,2] which simultaneously profiles gene expression and chromatin accessibility in the same cells, is a transformative technology for studying the landscape of gene regulation. Its unprecedented cellular resolution allows for the linking of *cis*-regulatory elements (CREs) to their target genes by calculating associations between the chromatin accessibility of CREs and the expression levels of target genes. These CRE-gene linkages characterize how gene expression levels are controlled and regulated by CREs in different tissues. Single-cell multi-omics sequencing has been widely applied to various species, tissue types, and disease conditions.[3,4] However, most studies focus only on a specific tissue and do not allow for direct comparisons of gene regulatory activities across tissues. Such comparative analysis is essential to identifying gene regulatory activities that are shared across or unique to certain tissue types, pinpointing how gene regulation drives the heterogeneity of tissue types.

RESULTS

Utilizing the massive amounts of publicly available data from single-cell multi-omics sequencing, we developed Compass for comparative analysis of gene regulation in single cells (Figure 1). Compass consists of two modules: a database, CompassDB, and an open-source R software package, CompassR. CompassDB is a large single-cell multi-omics database across various tissues and cell types. To build CompassDB, we downloaded 435 single-cell multi-omics samples from the ENCODE project[5] and Gene Expression Omnibus (GEO),[6] covering 41 human tissues and 23 mouse tissues (Figures 2A and 2B). We curated the metadata for each sample, including information on species, biological source (tissue or cell types),

This is an open access article under the CC BY license (http://creativecommons.org/licenses/by/4.0/).

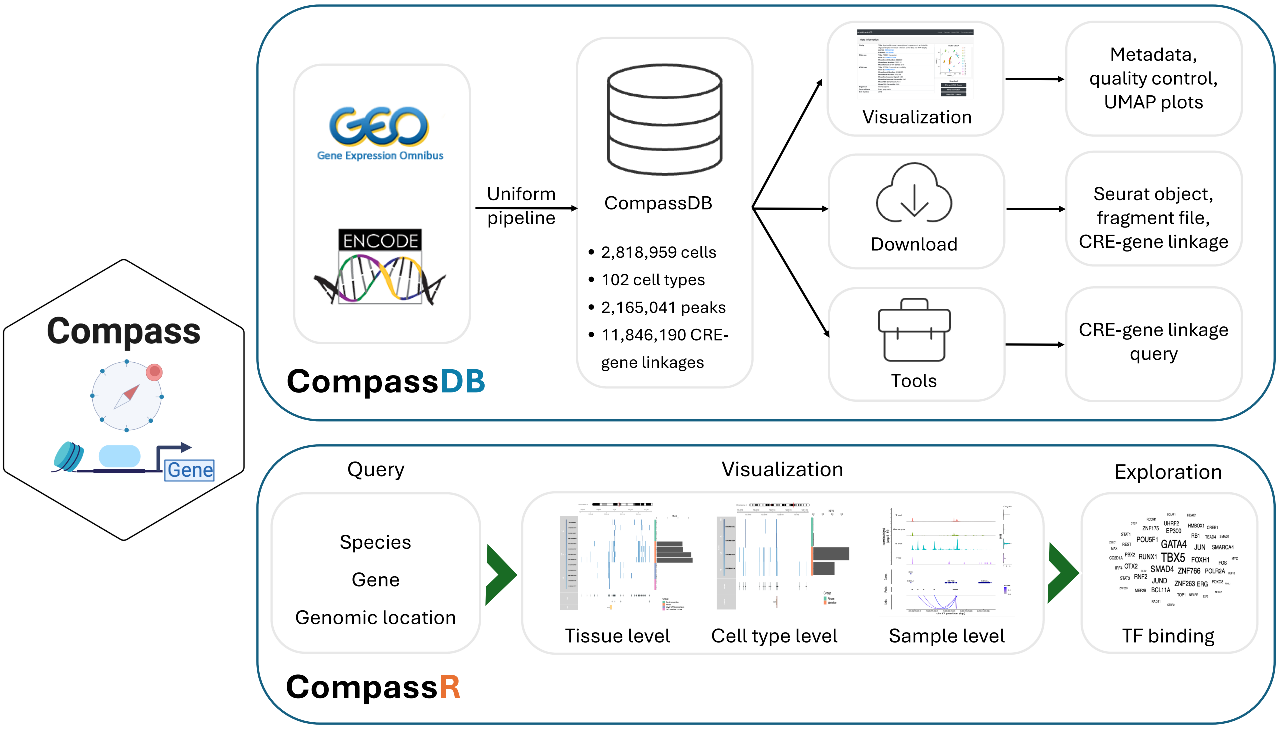

Figure 1. Schematic overview of Compass

age, gender, and disease status. We processed all samples with a uniform processing pipeline (STAR Methods) for gene expression and chromatin accessibility quantifications, quality control, peak calling, CRE-gene linkage, cell clustering, and cell type annotation. Both gene expression and chromatin accessibility profiles are separated by tissue types in a pseudobulk analysis (Figures S1A–S1D; STAR Methods), showing that the uniform processing pipeline is able to reliably recover the biological signals from the data. The processed data contain 2,818,959 single cells with high-quality gene expression and chromatin accessibility information, 41 unique tissue types, 102 unique cell types, 2,165,041 total peaks, and 11,846,190 pairs of CRE-gene linkages. Most CRE-gene linkages are present in only a very small number of tissues (Figures 2C and S2). For example, 61.0% of all CRE-gene linkages are found in only one tissue, and 95.0% occur in at most five tissues. This suggests that studying CRE-gene linkages in a limited number of tissues cannot fully capture their tissue specificity and may overlook important gene regulatory relationships. Therefore, a comparative analysis across various tissue types is essential to reconstruct the global gene regulatory landscape.

We developed an online web portal (http://compass-db.com/) for users to navigate and download processed and curated single-cell multi-omics samples. Users can search by keyword (Figure 2D) or browse a list of available cell types or tissue types (Figure 2E). For a selected sample, the web portal displays metadata information, quality control metrics, a uniform manifold approximation and projection (UMAP) plot, and a cell type annotation plot (Figure 2F). RNA and ATAC count matrices, ATAC fragment files, metadata information, and a list of CRE-gene linkages are available for download on the same page.

Compass enables the comparative analysis of gene regulation in two modes. In the first mode, a user can query a gene or a genomic region directly through the CompassDB online web portal (Figure 2G). The portal will then return a list of CRE-gene linkages associated with the queried gene or genomic region in all samples in the database. For each CRE-gene linkage, the list includes the linkage score and its p value obtained by Signac,[7] metadata information, motif information, and transcription factor (TF) binding activities through the Cistrome database.[8] This mode is useful when users want to quickly browse all biological contexts associated with the query. In the second mode, we developed CompassR to support more in-depth analyses and visualizations of CRE-gene linkages. Built upon CompassDB, CompassR can be used to analyze and visualize CRE-gene linkages in one or multiple samples and identify TFs whose binding is enriched in selected CREs (Figure 1). CompassR also allows users to incorporate additional single-cell multi-omics samples to enhance the analysis of gene regulation.

Figure 3A illustrates an example of CompassR analysis of the mouse Myh6 gene, which has important functions in cardiac muscle contraction and adult heart development.[9] We selected a group of samples from different mouse tissues, including gastrocnemius, heart, hippocampus, and cerebral cortex, for visualization. For each sample, CompassR visualizes the expression of Myh6 gene and the genomic locations of Myh6-linked CREs, whose chromatin accessibility is significantly associated with the gene expression of Myh6. CompassR also visualizes

CellPress
OPEN ACCESS

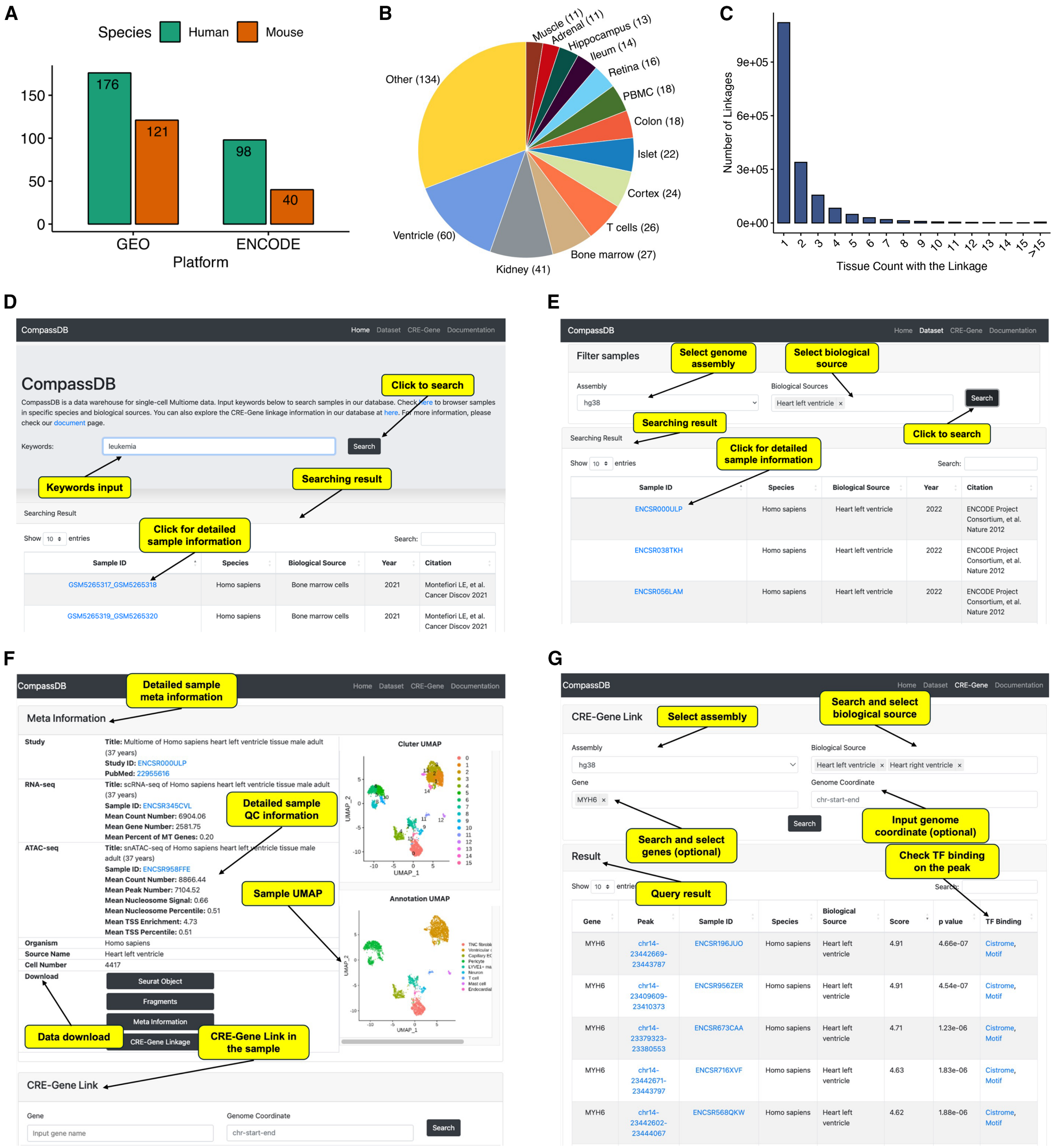

Figure 2. Overview of CompassDB

(A) Number of human and mouse samples collected in CompassDB from GEO and ENCODE.

(B) Names of tissues collected in CompassDB. Number of samples collected for each tissue is indicated in the parenthesis.

(C) Distribution of the number of tissues in which each CRE-gene linkage is present.

(D–G) Screenshots of CompassDB web portal showing keyword search page (D), sample search results (E), sample information page (F), and CRE-gene query page (G).

See also Figures S1 and S2.

Cell Reports Methods
Article

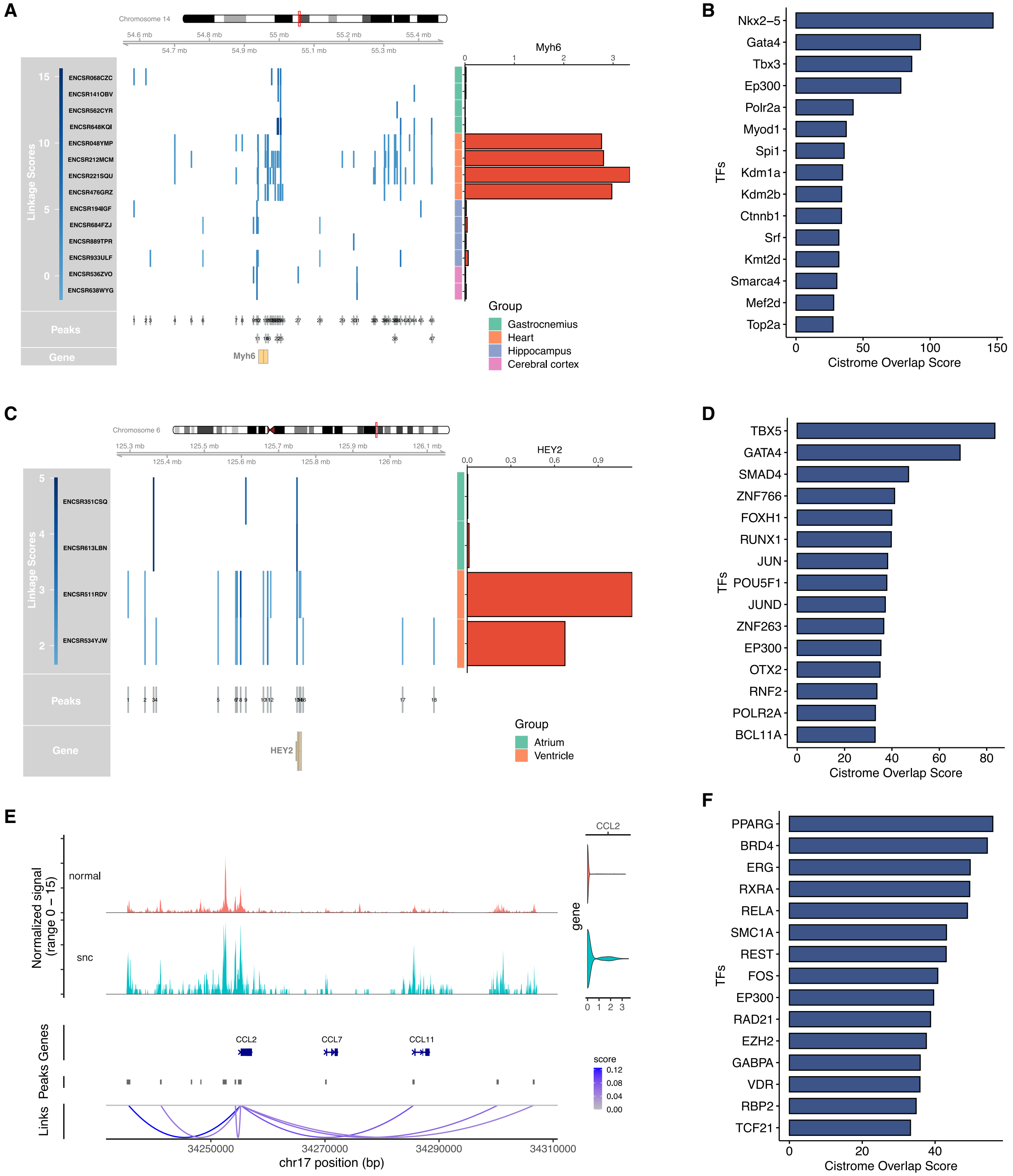

Figure 3. Application of CompassR for comparative gene regulation analysis

(A) CompassR analysis of Myh6 gene across four types of mouse tissues. The color in the heatmap shows CRE-gene linkage scores computed by Signac. The barplot shows mean expression of gene Myh6 in each sample.

(B) TFs enriched in heart-specific Myh6-linked CREs identified in (A).

(legend continued on next page)

the genomic locations of the Myh6 gene and a union set of Myh6-linked CREs across all samples.

CompassR identifies a larger number of Myh6-linked CREs that exist only in heart tissues. These CREs are reproducibly found across different heart samples, suggesting that they could be CREs that regulate the expression of the Myh6 gene only in the heart. It is worth noting that a study focusing solely on heart samples may identify the same set of CREs but cannot determine whether these regulatory behaviors are specific to the heart or generally present in other tissues. In contrast, CompassR identifies these regulations as heart specific by comparison across samples, which may explain the heterogeneous gene expression levels of Myh6 in the heart and other tissues. To better understand the mechanism driving the regulation, CompassR further identifies a list of TFs whose binding sites overlap with these heart-specific Myh6-linked CREs (STAR Methods; Figure 3B). Among them, Nkx2-5,[10] T-box TFs (Tbx),[11] Gata4,[12] and Srf[13] have been reported to regulate Myh6 in the heart.

In addition to studying whole tissues, CompassR can also study a specific cell type across tissues. For example, we compared the gene regulation of the HEY2 gene in cardiomyocytes between human atrium and ventricle tissues (Figure 3C). Cardiomyocytes are the muscle cells in the heart responsible for contracting and pumping blood. The atrium and ventricles contain cardiomyocytes with different functionalities and characteristics. HEY2 plays a crucial role in regulating the size of the cardiac progenitor pool,[14] the development of the right ventricle,[15] and the overall morphogenesis of the heart.[16] It has higher expression in ventricle cardiomyocytes.[14] For each sample, CompassR visualizes HEY2 gene expression levels in cardiomyocytes and HEY2-linked CREs found in cardiomyocytes that are significantly associated with HEY2 gene expression.

CompassR identifies many more HEY2-linked cardiomyocyte CREs in ventricle samples compared to atrium samples. These CREs are reproducibly found in the two ventricle samples and are, thus, likely ventricle-specific HEY2-linked CREs in cardiomyocytes, leading to the high expression levels of HEY2 genes in the ventricle cardiomyocytes. CompassR further identifies TFs whose binding sites overlap with these CREs (Figure 3D). Among them, TBX5[17] and GATA4[18] have been reported to regulate HEY2 expression during ventricle development.

Beyond analyzing multiple samples, CompassR can also visualize the details of gene expression, chromatin accessibility, and CRE-gene linkages in a single selected sample. Figure 3E demonstrates how CompassR can be used to analyze CRE-gene linkages during cellular senescence in stromal cells from a colon sample. Senescent cells were identified using DeepScence[19] software with its default settings. We focused on the CCL2 gene, a key marker of cellular senescence.[20] While the association between the CCL2 gene and cellular senescence has been reported, its gene regulatory mechanism in senescent versus normal cells remains largely unknown. For both senescent and normal cells, CompassR visualizes the aggregated chromatin accessibility in a genomic region flanking the CCL2 gene body, as well as the distribution of CCL2 gene expression levels. Additionally, CompassR displays the genomic locations of the CCL2 gene, the CREs, and the linkages between CREs and the CCL2 gene.

Compared to normal cells, gene expression and the chromatin accessibility levels of most CCL2-linked CREs are elevated in senescent cells, aligning with the function of CCL2 in cellular senescence. To further understand how CCL2 gene expression is regulated by CCL2-linked CREs, CompassR identifies TFs whose binding sites overlap with these CREs (Figure 3F). Among them, BRD4,[21] RELA,[22] EP300,[23] and EZH2[24] have been reported to play crucial roles in regulating cellular senescence, suggesting potential gene regulatory mechanisms of CCL2.

Additionally, we explored the regulation of the CD79B gene in a single peripheral blood mononuclear cell (PBMC) sample (Figure S3). CD79B is crucial for forming the B cell receptor complex and is expressed almost exclusively in B cells.[25] In the PBMC sample, we found that CD79B exhibits higher levels of gene expression and chromatin accessibility (Figure S3A). Four out of five CREs were associated with CD79B gene expression. Using CompassR, we identified EBF1,[26] SPI1,[27] SPIB,[28] RELA,[29] and PAX5,[30] which are enriched in CD79B-linked CREs and have been reported to play crucial roles in regulating B cell-specific genes, including CD79B (Figure S3B). These results suggest a potential mechanism for CD79B regulation.

DISCUSSION

In conclusion, Compass enables comparative analysis of gene regulation across samples from different tissues. Compass reveals tissue-specific CRE-gene linkages that cannot be found by studies focusing on a single tissue type, explaining the heterogeneity of gene expression across tissues. Additionally, CompassDB enables biologists to quickly explore gene regulatory activities associated with a given gene or genomic region. The massive amounts of uniformly processed single-cell multi-omics data provided by CompassDB also offer a valuable resource for building and benchmarking future computational methods. CompassDB will be updated regularly to incorporate new single-cell multi-omics samples, particularly those representing new tissue types or disease states.

Limitations of the study

The primary limitations of Compass stem from the nature of publicly available data. Despite our efforts to collect available

(C) CompassR analysis of HEY2 gene in cardiomyocytes, comparing atrium and ventricle. The color in the heatmap shows CRE-gene linkage scores computed by Signac. The barplot shows mean expression of gene HEY2 in each sample.

(D) TFs enriched in ventricle-specific HEY2-linked CREs in cardiomyocytes identified in (C).

(E) CompassR analysis of the CCL2 gene in stromal cells from a colon sample. The genome tracks display the normalized ATAC signals in normal and senescent (snc) stromal cells. Violin plots illustrate the expression of the CCL2 gene in each cell group. Arc plots depict CCL2-linked CREs, with colors indicating their linkage scores.

(F) TFs enriched in CCL2-linked CREs identified in (E).

See also Figure S3.

single-cell multi-omics datasets from numerous human and mouse tissues, the database does not encompass all possible tissue and cell types. Furthermore, the imbalance in the number of tissue types may introduce biases in the results. Although Compass employs a uniform data processing pipeline, batch effects from different studies may still bias the inferred gene regulatory patterns. Additional batch correction methods are needed to improve large-scale, cross-study comparisons. Finally, gene regulatory relationships in Compass are inferred through statistical associations. To establish the causal roles of CREs in gene regulation, further functional experiments are required.

RESOURCE AVAILABILITY

Lead contact

Requests for further information and resources should be directed to and will be fulfilled by the lead contact, Dr. Zhicheng Ji (zhicheng.ji@duke.edu).

Materials availability

This study did not generate new materials.

Data and code availability

- All data used in this study are available on the CompassDB website: http://compass-db.com.
- The CompassR package is available at GitHub: https://github.com/changxinw/CompassR and has also been deposited on Zenodo: https://doi.org/10.5281/zenodo.15170152. Code for reproducing the CompassR examples is provided in the package vignettes.
- Any additional information required to reanalyze the data reported in this paper is available from the lead contact upon request.

ACKNOWLEDGMENTS

C.W. and Z.J. are supported by the National Institutes of Health (NIH) Common Fund under award number U54AG075936 and by the NIH/National Institute of General Medical Sciences (NIGMS) under award number R35GM154865. W. H. is supported by the NIH/National Human Genome Research Institute (NHGRI) under award number R00HG011468 and by the NIH/NIGMS under award number R35GM150887.

AUTHOR CONTRIBUTIONS

Z.J. and C.W. conceived the study. C.W., H.M., M.C., W.H., and Z.J. contributed to the conceptualization and design of the method. C.W., Y.Q., Z.Y., and T.Z. conducted the analysis. C.W. and Z.J. wrote the manuscript.

DECLARATION OF INTERESTS

The authors declare no competing interests.

STAR★METHODS

Detailed methods are provided in the online version of this paper and include the following:

- KEY RESOURCES TABLE
- METHOD DETAILS
  - Data collection
  - Data processing
  - Pseudobulk analysis for quality control
  - Tissue specificity of CRE-gene linkages
  - CompassDB web portal
  - CompassR
- QUANTIFICATION AND STATISTICAL ANALYSIS

SUPPLEMENTAL INFORMATION

Supplemental information can be found online at https://doi.org/10.1016/j.crmeth.2025.101035.

Received: September 11, 2024
Revised: February 14, 2025
Accepted: April 14, 2025
Published: May 8, 2025

REFERENCES

1. Ma, S., Zhang, B., LaFave, L.M., Earl, A.S., Chiang, Z., Hu, Y., Ding, J., Brack, A., Kartha, V.K., Tay, T., et al. (2020). Chromatin potential identified by shared single-cell profiling of rna and chromatin. Cell *183*, 1103–1116.e20.
2. Chen, S., Lake, B.B., and Zhang, K. (2019). High-throughput sequencing of the transcriptome and chromatin accessibility in the same cell. Nat. Biotechnol. *37*, 1452–1457.
3. Han, M., Li, F., Zhang, Y., Dai, P., He, J., Li, Y., Zhu, Y., Zheng, J., Huang, H., Bai, F., and Gao, D. (2022). Foxa2 drives lineage plasticity and kit pathway activation in neuroendocrine prostate cancer. Cancer Cell *40*, 1306–1323.e8.
4. Wang, S.K., Nair, S., Li, R., Kraft, K., Pampari, A., Patel, A., Kang, J.B., Luong, C., Kundaje, A., and Chang, H.Y. (2022). Single-cell multiome of the human retina and deep learning nominate causal variants in complex eye diseases. Cell Genom. *2*, 100164.
5. Snyder, M.P., Gingeras, T.R., Moore, J.E., Weng, Z., Gerstein, M.B., Ren, B., Hardison, R.C., Stamatoyannopoulos, J.A., Graveley, B.R., et al.; ENCODE Project Consortium (2020). Perspectives on ENCODE. Nature *583*, 693–698.
6. Clough, E., and Barrett, T. (2016). The gene expression omnibus database. Methods Mol. Biol. *1418*, 93–110.
7. Stuart, T., Srivastava, A., Madad, S., Lareau, C.A., and Satija, R. (2021). Single-cell chromatin state analysis with signac. Nat. Methods *18*, 1333–1341.
8. Zheng, R., Wan, C., Mei, S., Qin, Q., Wu, Q., Sun, H., Chen, C.H., Brown, M., Zhang, X., Meyer, C.A., and Liu, X.S. (2019). Cistrome data browser: expanded datasets and new tools for gene regulatory analysis. Nucleic Acids Res. *47*, D729–D735.
9. Granados-Riveron, J.T., Ghosh, T.K., Pope, M., Bu'Lock, F., Thornborough, C., Eason, J., Kirk, E.P., Fatkin, D., Feneley, M.P., Harvey, R.P., et al. (2010). α-Cardiac myosin heavy chain (*MYH6*) mutations affecting myofibril formation are associated with congenital heart defects. Hum. Mol. Genet. *19*, 4007–4016.
10. Anderson, D.J., Kaplan, D.I., Bell, K.M., Koutsis, K., Haynes, J.M., Mills, R.J., Phelan, D.G., Qian, E.L., Leitoguinho, A.R., Arasaratnam, D., et al. (2018). Nkx2-5 regulates human cardiomyogenesis via a hey2 dependent transcriptional network. Nat. Commun. *9*, 1373.
11. Bruneau, B.G., Nemer, G., Schmitt, J.P., Charron, F., Robitaille, L., Caron, S., Conner, D.A., Gessler, M., Nemer, M., Seidman, C.E., and Seidman, J.G. (2001). A murine model of holt-oram syndrome defines roles of the t-box transcription factor tbx5 in cardiogenesis and disease. Cell *106*, 709–721.
12. Liang, Q., and Molkentin, J.D. (2002). Divergent signaling pathways converge on gata4 to regulate cardiac hypertrophic gene expression. J. Mol. Cell. Cardiol. *34*, 611–616.
13. Guo, Y., Jardin, B.D., Zhou, P., Sethi, I., Akerberg, B.N., Toepfer, C.N., Ai, Y., Li, Y., Ma, Q., Guatimosim, S., et al. (2018). Hierarchical and stage-specific regulation of murine cardiomyocyte maturation by serum response factor. Nat. Commun. *9*, 3837.
14. Gibb, N., Lazic, S., Yuan, X., Deshwar, A.R., Leslie, M., Wilson, M.D., and Scott, I.C. (2018). Hey2 regulates the size of the cardiac progenitor pool during vertebrate heart development. Development *145*, dev167510.

CellPress
OPEN ACCESS

15. Seya, D., Ihara, D., Shirai, M., Kawamura, T., Watanabe, Y., and Nakagawa, O. (2021). A role of hey2 transcription factor for right ventricle development through regulation of tbx2-mycn pathway during cardiac morphogenesis. Dev. Growth Differ. *63*, 82–92.
16. Donovan, J., Kordylewska, A., Jan, Y.N., and Utset, M.F. (2002). Tetralogy of fallot and other congenital heart defects in hey2 mutant mice. Curr. Biol. *12*, 1605–1610.
17. Watanabe, Y., Wang, Y., Tanaka, Y., Iwase, A., Kawamura, T., Saga, Y., Yashiro, K., Kurihara, H., and Nakagawa, O. (2023). *Hey2* enhancer activity defines unipotent progenitors for left ventricular cardiomyocytes in juxtacardiac field of early mouse embryo. Proc. Natl. Acad. Sci. USA *120*, e2307658120.
18. Ihara, D., Watanabe, Y., Seya, D., Arai, Y., Isomoto, Y., Nakano, A., Kubo, A., Ogura, T., Kawamura, T., and Nakagawa, O. (2020). Expression of hey2 transcription factor in the early embryonic ventricles is controlled through a distal enhancer by tbx20 and gata transcription factors. Dev. Biol. *461*, 124–131.
19. Qu, Y., Dong, R., Gu, L., Chan, C., Xie, J., Glass, C., Wang, X.F., Nixon, A. B., and Ji, Z. (2024). Single-cell and spatial detection of senescent cells using deepscence. Preprint at bioRxiv. https://doi.org/10.1101/2023.11.21.568150.
20. Liu, Y., Pan, J., Pan, X., Wu, L., Bian, J., Lin, Z., Xue, M., Su, T., Lai, S., Chen, F., et al. (2019). Klotho-mediated targeting of ccl 2 suppresses the induction of colorectal cancer progression by stromal cell senescent microenvironments. Mol. Oncol. *13*, 2460–2475.
21. Tasdemir, N., Banito, A., Roe, J.S., Alonso-Curbelo, D., Camiolo, M., Tschaharganeh, D.F., Huang, C.H., Aksoy, O., Bolden, J.E., Chen, C.C., et al. (2016). Brd4 connects enhancer remodeling to senescence immune surveillance. Cancer Discov. *6*, 612–629.
22. Wang, J., Jacob, N.K., Ladner, K.J., Beg, A., Perko, J.D., Tanner, S.M., Liyanarachchi, S., Fishel, R., and Guttridge, D.C. (2009). Rela/p65 functions to maintain cellular senescence by regulating genomic stability and dna repair. EMBO Rep. *10*, 1272–1278.
23. Sen, P., Lan, Y., Li, C.Y., Sidoli, S., Donahue, G., Dou, Z., Frederick, B., Chen, Q., Luense, L.J., Garcia, B.A., et al. (2019). Histone acetyltransferase p300 induces de novo super-enhancers to drive cellular senescence. Mol. Cell *73*, 684–698.e8.
24. Fan, T., Jiang, S., Chung, N., Alikhan, A., Ni, C., Lee, C.C.R., and Hornyak, T.J. (2011). Ezh2-dependent suppression of a cellular senescence phenotype in melanoma cells by inhibition of p21/cdkn1a expression. Mol. Cancer Res. *9*, 418–429.
25. Chu, P.G., and Arber, D.A. (2001). Cd79: a review. Appl. Immunohistochem. Mol. Morphol. *9*, 97–106.
26. Györy, I., Boller, S., Nechanitzky, R., Mandel, E., Pott, S., Liu, E., and Grosschedl, R. (2012). Transcription factor ebf1 regulates differentiation stage-specific signaling, proliferation, and survival of b cells. Genes Dev. *26*, 668–682.
27. DeKoter, R.P., and Singh, H. (2000). Regulation of b lymphocyte and macrophage development by graded expression of pu. 1. Science *288*, 1439–1441.
28. Willis, S.N., Tellier, J., Liao, Y., Trezise, S., Light, A., O'Donnell, K., Garrett-Sinha, L.A., Shi, W., Tarlinton, D.M., and Nutt, S.L. (2017). Environmental sensing by mature b cells is controlled by the transcription factors pu. 1 and spib. Nat. Commun. *8*, 1426.
29. Almalki, W.M. (2021). Role of Traf Family of Proteins in Mediating CD40 Stimulation-Induced Drug Resistance in Chronic Lymphocytic Leukaemia Cells (The University of Liverpool).
30. Nutt, S.L., Urbánek, P., Rolink, A., and Busslinger, M. (1997). Essential functions of pax5 (bsap) in pro-b cell development: difference between fetal and adult b lymphopoiesis and reduced v-to-dj recombination at the igh locus. Genes Dev. *11*, 476–491.
31. Davis, S., and Meltzer, P.S. (2007). Geoquery: a bridge between the gene expression omnibus (geo) and bioconductor. Bioinformatics *23*, 1846–1847.
32. Hao, Y., Stuart, T., Kowalski, M.H., Choudhary, S., Hoffman, P., Hartman, A., Srivastava, A., Molla, G., Madad, S., Fernandez-Granda, C., and Satija, R. (2024). Dictionary learning for integrative, multimodal and scalable single-cell analysis. Nat. Biotechnol. *42*, 293–304.
33. Li, M., Zhang, X., Ang, K.S., Ling, J., Sethi, R., Lee, N.Y.S., Ginhoux, F., and Chen, J. (2022). Disco: a database of deeply integrated human single-cell omics data. Nucleic Acids Res. *50*, D596–D602.
34. Wang, C., Sun, D., Huang, X., Wan, C., Li, Z., Han, Y., Qin, Q., Fan, J., Qiu, X., Xie, Y., et al. (2020). Integrative analyses of single-cell transcriptome and regulome using maestro. Genome Biol. *21*, 198.

STAR★METHODS

KEY RESOURCES TABLE

REAGENT or RESOURCE	SOURCE	IDENTIFIER
Deposited data		
CompassDB	Open source	http://compass-db.com/
Software and algorithms		
R (version 4.1.2)	Open source	https://www.r-project.org/
GEOquery (version 2.62.2)	Open source	https://www.bioconductor.org/packages/release/bioc/html/GEOquery.html
Cell Ranger ARC software (version 323 2.0.1)	Open source	https://www.10xgenomics.com/support/software/cell-ranger-arc/latest
Seurat (version 4.3.0)	Open source	https://satijalab.org/seurat/
Signac (version 1.8.0)	Open source	https://stuartlab.org/signac/
CompassR (version 1.0.0)	This study	https://doi.org/10.5281/zenodo.15170151
Other		
GEO	Open source	https://www.ncbi.nlm.nih.gov/geo/
ENCODE	Open source	https://www.encodeproject.org/
DISCO	Open source	https://www.immunesinglecell.org/

METHOD DETAILS

Data collection

Samples in this study were collected from GEO[6] and the ENCODE project.[5] To search for single-cell multi-omics samples in GEO, we queried the GEO website using the keyword “Multiome” and constrained the species to human and mouse. The query was performed on June 24th, 2024. For each sample, its raw sequencing files in FASTQ format were downloaded from SRA. Metadata information for samples from GEO was queried through the R package GEOquery (version 2.62.2).[31] All single-cell Multiome samples available on the ENCODE portal (https://www.encodeproject.org/) were included in this study. The query was performed on October 14th, 2022. For each sample, its raw sequencing files in FASTQ format and its metadata information were directly obtained from the ENCODE portal.

Data processing

Read alignment

For each sample, FASTQ files were processed with 10x Cell Ranger ARC software (version 2.0.1) to align the reads to the human GRCh38 or mouse mm10 genome (10x version 2020-A-2.0.0). Cell Ranger ARC produced a gene expression count matrix for the RNA modality and a fragments file for the ATAC modality. Each sample was then processed separately using the following pipeline.

Cell filtering

Cells that met the following six criteria were retained for further processing: number of RNA reads greater than 1,000; number of RNA reads fewer than 25,000; number of ATAC reads greater than 1,000; number of ATAC reads fewer than 100,000; nucleosome signal (calculated by Signac’s `NucleosomeSignal` function) less than 2; and TSS enrichment score (calculated by Signac’s `TSSEnrichment` function) greater than 1.

Processing RNA modality

For the RNA modality, Seurat (version 4.3.0)[32] was used to further process the gene expression count matrix. Specifically, the count matrix was library size normalized and log-transformed (log-normalized) using the function `NormalizeData` with a scale factor of 10,000. The top 2000 variable features were selected by the function `FindVariableFeatures`. The gene expression matrix was then scaled using the `ScaleData` function. Dimensionality reduction with principal component analysis (PCA) was performed using the `RunPCA` function on the expression of the top 2000 variable genes.

Processing ATAC modality

For the ATAC modality, Signac (version 1.8.0)[7] was used to further process the fragments file from Cellranger ARC. Specifically, peak calling was performed with the `CallPeaks` function across all cells in the sample. Non-standard chromosomes were filtered using

the keepStandardChromosomes function. Blacklist regions were removed by subsetByOverlaps with the genome blacklist provided by Signac. The filtered peaks are referred to as "CREs" throughout this study.

A peak-level chromatin accessibility count matrix was then obtained using the FeatureMatrix function. Top features were selected using FindTopFeatures with the min.cutoff parameter set to 5. The count matrix was then normalized using TF-IDF with the RunTFIDF function, and iterative latent semantic indexing (LSI) was performed using the RunSVD function.

In addition to sample-level peaks, cell-type-level peaks were also called using the CallPeaks function with the group.by parameter set to the annotated cell types obtained in the following step.

Integration of RNA and ATAC modalities

To integrate information from the RNA and ATAC modalities, we used Seurat's FindMultiModalNeighbors function with PCA dimensions 1 to 50 and LSI dimensions 2 to 40 as the input to construct a weighted nearest neighbor (WNN) graph. The WNN was used to perform cell clustering using the Louvain algorithm (FindClusters function) with a resolution of 1. The low-dimensional representation was further obtained using UMAP (RunUMAP function) with the WNN as input. Finally, CRE-gene linkage was computed using Signac's LinkPeaks function. This function only considers CREs within 50,000 base pairs (bp) upstream or downstream of a gene's transcription start site (TSS).

Cell type annotation

Cell type annotation was performed for each cell cluster in each sample. To annotate the cell type of a cell cluster, we compared its aggregated gene expression profile with those obtained from DISCO,[33] a large database containing preprocessed single-cell RNA-seq data from hundreds of tissues and cell lines.

For each sample, we first identified its corresponding tissue type in the DISCO database and downloaded the log-normalized single-cell gene expression matrix for that tissue type from DISCO. We then created DISCO pseudobulks by aggregating gene expression profiles across cells within each cell type using Seurat's AverageExpression function. Likewise, we also created multiome pseudobulks for single-cell multi-omics data by aggregating log-normalized gene expression profiles across cells within each cell cluster. Spearman correlation coefficients were computed between each DISCO pseudobulk and each multiome pseudobulk using the top 3000 most variable genes. For each multiome pseudobulk, the cell type name of the DISCO pseudobulk that has the highest correlation coefficient with the multiome pseudobulk was assigned to the multiome cell cluster as the annotated cell type.

For samples from cell lines, sorted cell types, or tissues whose counterparts cannot be found in the DISCO database, the cells were annotated with the original names of the cell line, cell type, or tissue.

Pseudobulk analysis for quality control

Pseudobulk analysis was performed for RNA and ATAC modalities and for human and mouse separately. To obtain a pseudobulk feature matrix for the RNA modality, we computed a pseudobulk for each sample by aggregating the log-normalized gene expression levels across all cells in that sample using Seurat's AverageExpression function. To obtain a pseudobulk feature matrix for the ATAC modality, the genome was segmented into 100,000 bp non-overlapping bins. For each sample and each bin, we calculated the total number of reads from all cells in that sample that overlap with that bin. The bin-level count matrix was then log-normalized using Seurat's NormalizeData function.

For each pseudobulk feature matrix obtained from either RNA or ATAC, the top 10,000 most variable features were selected using Seurat's FindVariableFeatures function. These top variable features were further visualized using the pheatmap (version 1.0.12) R package, after scaling each feature across pseudobulks to have a mean of 0 and a standard deviation of 1.

Tissue specificity of CRE-gene linkages

Peaks across all human samples were merged to form a union set of CREs. For each original CRE-gene linkage, the CRE was replaced with the overlapping CRE from the union set to ensure comparability across samples. The number of human tissues in which each CRE-gene linkage is present was then calculated and visualized in Figure 2C. Figure S2 illustrates the linkage strength, calculated using Signac's LinkPeaks function, for CRE-gene linkages present in a tissue.

CompassDB web portal

Implementation

The CompassDB website is composed of a back-end built with the Django framework and a front-end user interface built with HTML and JavaScript. User inputs received by the front-end are passed to the back-end. The back-end then queries a MySQL database and returns the information to the front-end using an application programming interface (API).

Search for samples

CompassDB supports two modes to search for single-cell multi-omics samples of interest. In the first mode, users can query CompassDB by keywords. The keywords can be related to any information about the samples, such as species or the name of the publication. In the second mode, users can query CompassDB by selecting the species and the name of the tissue or cell type. For both modes, CompassDB will return a list of samples meeting the search criteria and their metadata information. Clicking on the name of a sample will lead the user to a new sample information page, which will be discussed below.

Sample information page

The sample information page contains five types of information: metadata, including the name of the study, species, and tissue or cell type name; RNA and ATAC quality control metrics, such as the average number of reads and the average number of peaks; UMAP plots showing the cell clusters and annotated cell types; URL links for downloading RNA and ATAC read count matrices, metadata information of the sample, ATAC fragment file, and CRE-gene linkage results; and an interactive, searchable table for querying CRE-gene linkages associated with a specific gene or genomic region in the sample.

Search for CRE-gene linkage across samples

In addition to searching for CRE-gene linkages within one sample, CompassDB also allows searching for CRE-gene linkages across some or all samples in the database. Users need to specify a gene or genomic region of interest and can optionally specify only samples with the desired species or tissue or cell type to be included in the returned results. If a gene is specified as input, CompassDB will return all CRE-gene linkages associated with that gene. If a genomic region is specified as input, CompassDB will find all CREs overlapping with the given genomic region and return all CRE-gene linkages in all samples associated with any of the overlapping CREs.

For each CRE-gene linkage, CompassDB returns the following information: the gene name, the genomic location of the CRE, the clickable name of the sample, species, tissue or cell type name, linkage score and its p-value calculated by Signac's `LinkPeaks` function, TF binding activities through the Cistrome database,[8] and the IDs, names, and scores of motifs in the CRE computed by the `FindMotifs` function provided by Signac.[7]

CompassR

Mode 1: Comparative analysis of gene regulation across tissues

In this mode, the user needs to first select a gene and a set of samples of interest. For each selected sample, CompassR retrieves all CRE-gene linkages associated with the selected gene from CompassDB. A CRE that is involved in a CRE-gene linkage is called a gene-linked CRE. CompassR then uses a heatmap to visualize the genomic locations of all gene-linked CREs in all selected samples. The genomic location of the selected gene and a union set of all gene-linked CREs (union gene-linked CREs) are visualized at the bottom of the heatmap. The mean gene expression level of the selected gene in each selected sample is visualized to the right of the heatmap.

The user can further study TFs associated with tissue-specific gene-linked CREs. For a tissue of interest selected by the user, an element in the set of union gene-linked CREs is considered a tissue-specific gene-linked CRE if it overlaps with gene-linked CREs in more than half of the samples from the selected tissue and in less than half of the samples from any other tissue. CompassR uses the `ATACAnnotateTranscriptionFactor` function in MAESTRO[34] to identify enriched TFs in these tissue-specific gene-linked CREs. Briefly, the function searches Cistrome DB[8] for ChIP-seq samples whose peaks overlap with the input CREs and returns the corresponding TFs and binding scores of the samples.

Mode 2: Comparative analysis of gene regulation across tissues and within a cell type

This mode is similar to the first mode except that the user needs to additionally select a cell type of interest. In addition to being involved in a CRE-gene linkage, a CRE in a sample must also overlap with cell-type-level ATAC peaks of the selected cell type in that sample to be considered a gene-linked CRE in the selected cell type. Once the gene-linked CREs are obtained, all subsequent analyses are the same as in the first mode.

Mode 3: Comparative analysis of gene regulation within a sample

In this mode, the user needs to select a gene and a sample of interest. CompassR then visualizes four components in the same plot using Signac's `CoveragePlot` function. The first component is the normalized signals of chromatin accessibility for each cell type in a flanking region (10,000 bp upstream and downstream by default) of the selected gene. The second component is the distribution of single-cell log-normalized gene expression levels within each cell type. The third component is the genomic locations of the selected gene, nearby genes, and CREs. The fourth component is the CRE-gene linkages associated with the selected gene. Similar to the first mode, CompassR uses Cistrome to identify enriched TFs in the gene-linked CREs.

QUANTIFICATION AND STATISTICAL ANALYSIS

All statistical analyses were performed using R (version 4.1.2). For processing single-cell multi-omics data, we used Seurat (version 4.3.0) and Signac (version 1.8.0). For chromatin accessibility and gene expression data, normalization, variable feature selection, dimensionality reduction, clustering, and visualization were conducted using standard Seurat and Signac pipelines, as detailed in the Data Processing section. CRE-gene linkages were identified using Signac's `LinkPeaks` function, which calculates linkage scores and p-values in Figures 2G, 3A, 3C, 3E, and S3A.

The number of replicates (n) corresponds to the number of samples in Figures 3A, 3C, S1, and S2. Pseudobulk analyses were performed by aggregating log-normalized gene expression or TF-IDF-normalized chromatin accessibility values across all cells within a sample in Figure S1. All statistical tests and significance values are detailed in the figure legends. No data were excluded unless specified.

Cell Reports Methods, Volume *5*

Supplemental information

Comparative analysis of gene regulation

in single cells using Compass

Changxin Wan, Yilong Qu, Zhiyou Ye, Tianbei Zhang, Huifang Ma, Ming Chen, Wenpin Hou, and Zhicheng Ji

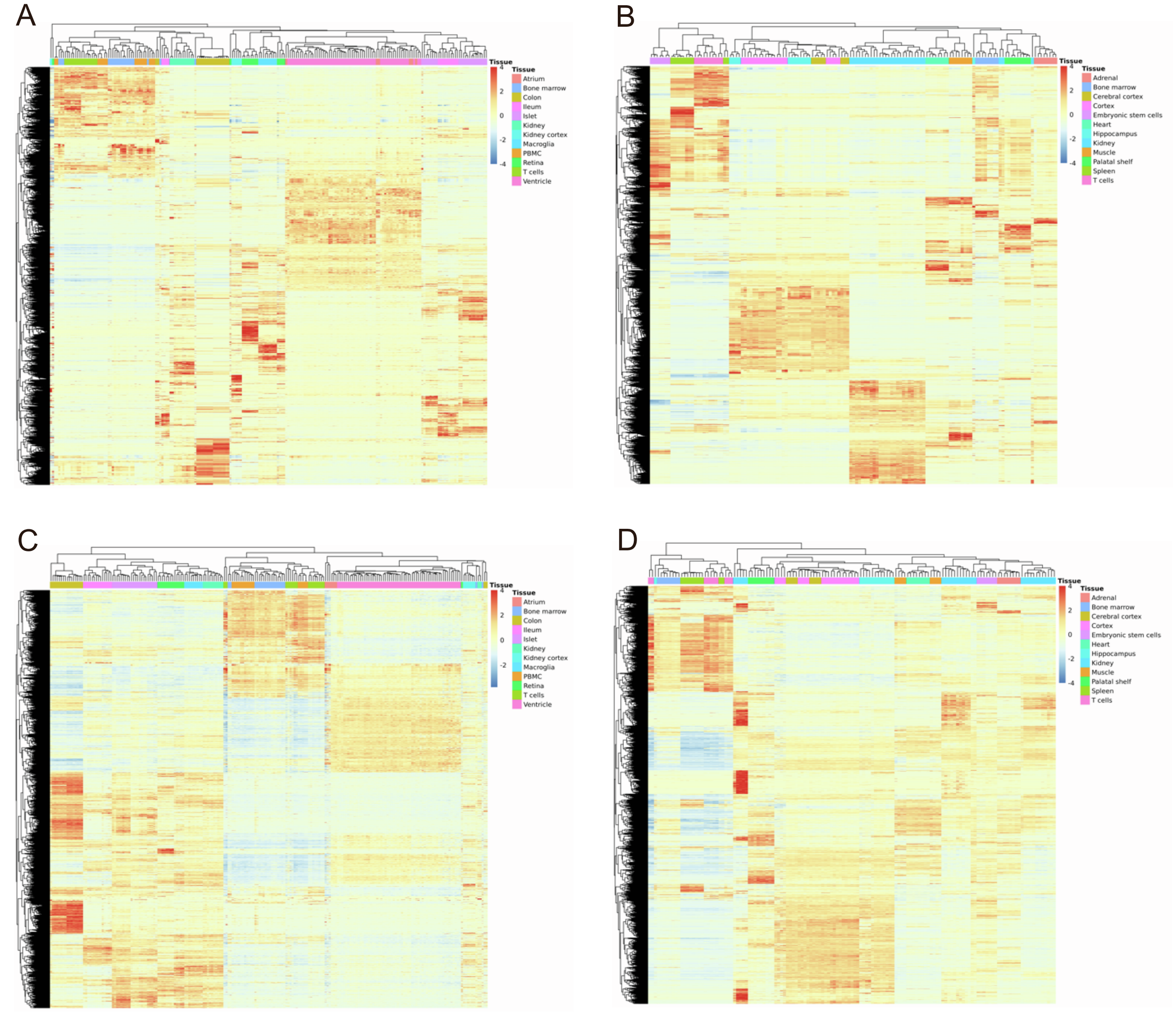

Figure S1. Pseudobulk analysis in CompassDB, related to Figure 2.

(A - B) Gene expression levels of RNA pseudobulks in human (A) and mouse (B) samples. Each row represents a gene and each column represents a sample. Gene expression values are scaled to have a mean of 0 and a standard deviation of 1 for each gene across samples. Samples from different tissues are marked in different colors.

(C - D) Chromatin accessibility levels of ATAC pseudobulks in human (C) and mouse (D) samples. Each row represents a genomic bin and each column represents a sample. Chromatin accessibility values are scaled to have a mean of 0 and a standard deviation of 1 for each genomic bin across samples. Samples from different tissues are marked in different colors.

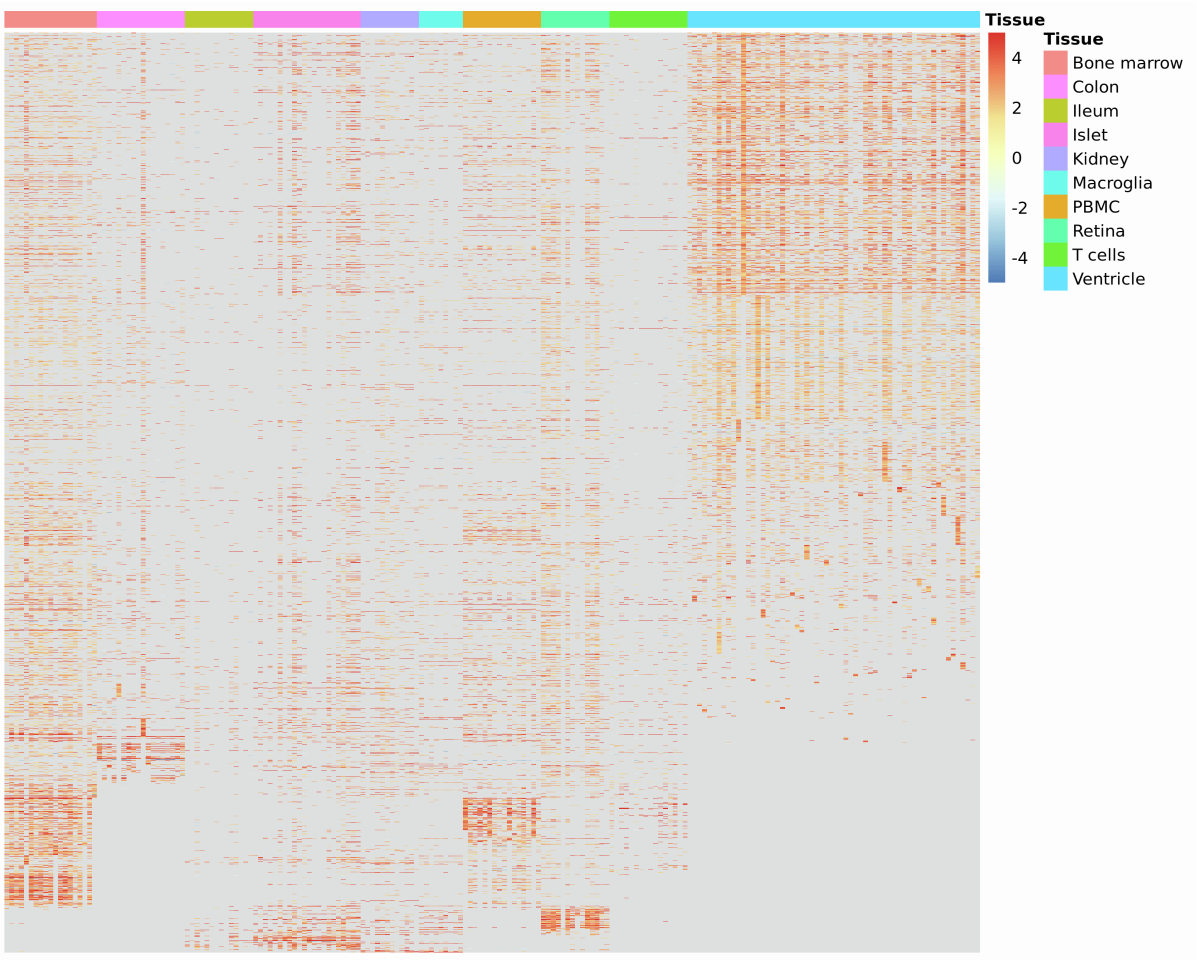

Figure S2. CRE-Gene linkage signal in human samples, related to Figure 2. Each row represents a CRE-gene linkage, and each column represents a sample. If a linkage is present in a tissue, the color indicates the linkage strength values from Signac. Linkages not present in a tissue are shown in gray.

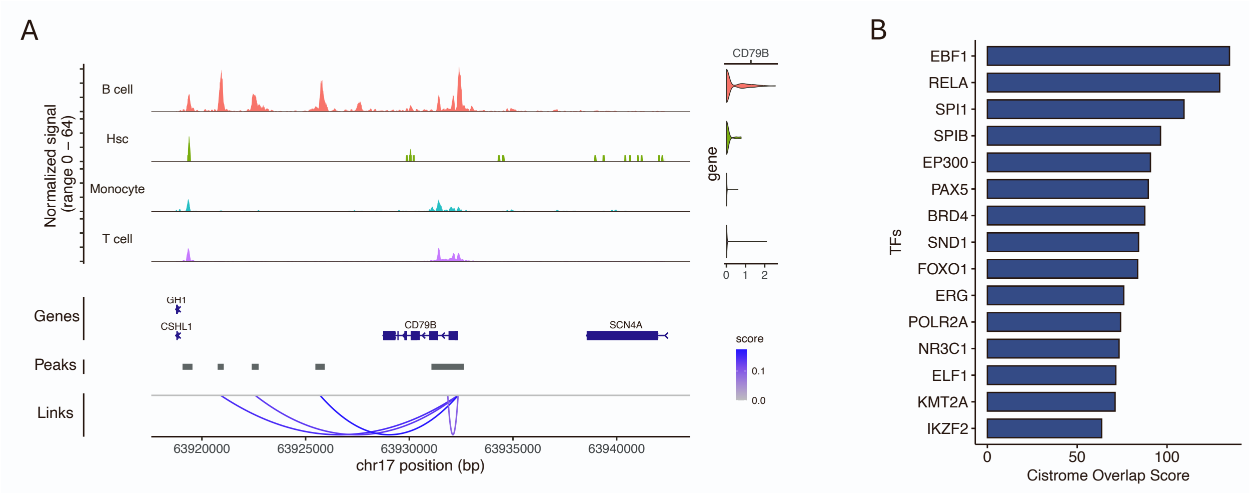

Figure S3. CompassR analysis of the CD79B gene in PBMCs, related to Figure 3. The genome tracks show the normalized ATAC signals in each cell type. Violin plots show expression of CD79B gene in each cell type. Arc plots show CD79B-linked CREs and the color indicates their linkage scores.

(A) CompassR analysis of CD79B gene in a PBMC sample.

(B) TFs enriched in CD79B-linked CREs identified in (A).
